# Leaf Epiphytic Bacteria of Plants Colonizing Mine Residues: Possible Exploitation for Remediation of Air Pollutants

**DOI:** 10.3389/fmicb.2018.03028

**Published:** 2018-12-07

**Authors:** Ariadna S. Sánchez-López, Ma. del Carmen A. González-Chávez, Fernando A. Solís-Domínguez, Rogelio Carrillo-González, Greta H. Rosas-Saito

**Affiliations:** ^1^Bio-Engineering Laboratory, Facultad de Ingeniería, Universidad Autónoma de Baja California, Mexicali, Mexico; ^2^Soil and Environmental Chemistry Laboratory, Edaphology Program, Colegio de Postgraduados, Texcoco, Mexico; ^3^Electron Microscopy Laboratory, Red de Estudios Moleculares Avanzados, Instituto Nacional de Ecología, Xalapa Enríquez, Mexico

**Keywords:** phytoremediation, phylloremediation, particulate matter, air pollutants, heavy metals

## Abstract

Plant surfaces are known as an important sink for various air pollutants, including particulate matter and its associated potentially toxic elements (PTE). Moreover, leaves surface or phylloplane is a habitat that harbors diverse bacterial communities (epiphytic). However, little is known about their possible functions during phytoremediation of air pollutants like PTE. The study of leaf epiphytic bacteria of plants colonizing mine residues (MR) containing PTE is thus a key to understand and exploit plant–epiphytic bacteria interactions for air phytoremediation purposes. In this research, we aimed (i) to characterize the functions of epiphytic bacteria isolated from the phylloplane of *Brickellia veronicifolia*, *Flaveria trinervia*, *Gnaphalium* sp., and *Allionia* *choisyi* growing spontaneously on multi-PTE contaminated MR and (ii) to compare these against the same plant species in a non-polluted control site (NC). Concentrations (mg kg^-1^) of PTE on MR leaf surfaces of *A. choisyi* reached up to 232 for Pb, 13 for Cd, 2,728 for As, 52 for Sb, 123 for Cu in *F. trinervia*, and 269 for Zn in *Gnaphalium* sp. In the four plant species, the amount of colony-forming units per cm^2^ was superior in MR leaves than in NC ones, being *A. choisyi* the plant species with the highest value. Moreover, the proportion of isolates tolerant to PTE (Zn, Cu, Cd, and Sb), UV light, and drought was higher in MR leaves than in those in NC. Strain BA15, isolated from MR *B. veronicifolia*, tolerated 150 mg Zn L^-1^, 30 mg Sb L^-1^, 25 mg Cu L^-1^; 80 mg Pb L^-1^, and was able to grow after 12 h of continuous exposition to UV light and 8 weeks of drought. Plant growth promotion related traits [N fixation, indole acetic acid (IAA) production, and phosphate solubilization] of bacterial isolates varied among plant species isolates and between MR and NC sampling condition. The studied epiphytic isolates possess functions interesting for phytoremediation of air pollutants. The results of this research may contribute to the development of novel and more efficient inoculants for microbe-assisted phytoremediation applied to improve air quality in areas exposed to the dispersion of metal mine tailings.

## Introduction

The atmospheric dispersion of different pollutants has become a concern due to the consequences caused in the public health and the environment. Potentially toxic elements (PTE) associated with solid particles are among these pollutants. Mine tailings generally represent an environmental issue due to the high concentrations of PTE that are commonly found in this kind of material (Alloway, 2013). PTE can be easily dispersed in the environment through the action of wind (aerial dispersion of solid particles) or water (runoffs and percolation) (González and González-Chávez, 2006; Cidu, 2011; García-Lorenzo et al., 2012). In arid and semi-arid regions, the restrictive environmental conditions limit the development of a plant cover; thus, MR remain exposed to the action of environment promoting dispersion of particles containing PTE (Mendez and Maier, 2008). Plant surfaces are recognized as a significant sink for a wide diversity of pollutants (Dzierżanowski and Gawroński, 2011; Bamniya et al., 2012; Mao et al., 2012; Sæbø et al., 2012; Ram et al., 2014). Recently, the role of the pioneer plants colonizing MR in the reduction of atmospheric dispersion of PTE associated with solid particles was documented and named as phytobarrier (Sánchez-López et al., 2015a).

Some authors have pointed out that a convenient selection of plants for phytoremediation of PTE-associated particles includes: besides trees, the presence of shrubs, subshrubs, and herbaceous vegetation with dense foliage close the ground, mixture of evergreen and deciduous plants, and use of indigenous fast-growing plants (Jamil et al., 2009; Weber et al., 2014). Certainly, the inclusion of plant species able to retain high amounts of particles is also necessary. In this research, we study four different plant species: *Brickellia veronicifolia*, *Flaveria trinervia*, *Gnaphalium* sp., and *Allionia choisyi*. These species were previously investigated and reported with high capacity to retain solid particles associated with PTE (Sánchez-López et al., 2015a). They are commonly found spontaneously colonizing MR containing diverse PTE (Sánchez-López et al., 2015b) and represent a mixture of plants: shrubs (*B. veronicifolia*), herbaceous (*F. trinervia* and *Gnaphalium* sp.), and creeping species (*A. choisyi*); moreover, some are evergreen (*B. veronicifolia* and *Gnaphalium* sp.) and others are deciduous (*F. trinervia* and *A. choisyi*). Therefore, the suggested characteristics for a suitable vegetated cover that diminishes the dispersion of particles are covered.

Even though several studies describe the ability of plants to retain PTE-containing particles on their leaves surface, the possible benefits that associated microorganisms, specifically epiphytic microorganisms, offer to their host plants should be taken into account too. A diverse and abundant community of microorganisms naturally exists in the above-ground portions of plants (Vorholt, 2012). It is one of the most common microbial habitats on earth and bacteria are by far the most abundant and constant members (Lindow and Brandl, 2003; Vorholt, 2012). As described for other plant-associated bacteria, the close connection between plant species and microbial communities in the leaf environment suggests their adaptation and co-evolutionary relationships (Wei et al., 2017). In the case of rhizospheric and endophytic bacteria, these associations have been widely studied and exploited for their application in the phytoremediation of soil and water polluted by PTE (Weyens et al., 2009; Rajkumar et al., 2012; Ma et al., 2016). However, much less is known about plant-epiphytic microorganisms. Since epiphytic microorganisms are found on plant surfaces, they remain exposed to adverse conditions like UV radiation, desiccation, nutrient scarcity, wind, wide diversity of pollutants, and many other biotic and abiotic factors (Thapa et al., 2017). It is mentioned that a long-lasting exposure of leaves and leaf-associated microbes to air pollutants may result in the development of mechanisms for adapting to the contaminants and specific environment conditions (Wei et al., 2017); thus, exploring plant-epiphytic microbes become of relevance for its possible application for phytoremediation of airborne pollutants (Weyens et al., 2015; Wei et al., 2017).

Therefore, this research aimed to study the functions of epiphytic bacteria isolated from the phylloplane of four different plant species (*B. veronicifolia, F. trinervia, Gnaphalium* sp., and A. *choisyi*) acting as phytobarriers on multi-PTE contaminated mine tailings and compare them against those from control (non-polluted) site.

## Materials and Methods

### Plant Sampling and Locations

The site of the study was the mining area of Zimapan, located in the central part of Mexico. The climate is semi-arid. Sampling was done during September 2017, the average temperature of the month is 20°C, with maximum and minimum of 30 and 11.5°C, respectively. Precipitation of the month was 84 mm (SMN, 2017). The selection of plant species was made according to their capacity to retain PTE-containing particles (phytobarriers) previously reported by Sánchez-López et al. (2015a). The studied species were as follows: *B. veronicifolia*, *F. trinervia*, *Gnaphalium* sp., and *A. choisyi*. Five leaf composed samples of the four plant species were taken from two conditions: pollution by PTE (MR) and without pollution (control site; NC). Leaves from different size and without visible disease symptoms or physical damage were collected; too old and too young leaves were avoided. Leaves were randomly taken in order to obtain a representative composed sample. For PTE-polluted condition, all four species were found in the same mine tailing Santa Maria (20°44′8.89″N, 99°23′56.07″W). In the case of the control condition, sampling at different sites was needed to find the studied plant species. *B. veronicifolia* and *A. choisyi* were found together approximately 5.3 km away from the MR (20°44′16.084″N, 99°21′59.14″W), whereas *F. trinervia* and *Gnaphalium* sp. plants were located about 3 km from the mine (20°44′6.49″N, 99°22′30.593″W).

### PTE Concentrations in the Rhizosphere and on Leaf Surfaces

The total concentrations of PTE in rhizosphere were determined with HNO_3_ using the USEPA 3051 method microwave assisted digestion (Environmental Protection Agency [EPA], 1994), 5 g of air-dried sieved (2 mm) soil were taken for the analysis. To estimate the metal concentration on leaves surfaces, the methodology reported by Sánchez-López et al. (2015a) was applied. In brief, plant samples were divided into two parts, one part was kept as collected in the field skipping washing procedure and the other one was thoroughly washed. All samples were dried at 65°C until constant weight and then milled. 0.5 g of powdered leaves were microwave digested in HNO_3_ according to EPA 3051. Digests were replenished up to 25 mL, for plant samples, and 50 mL for soil samples, and then filtered (Whatman 42). Concentrations of Zn, Cd, Pb, Ni, Cu, As, and Sb in each digest were determined using flame atomic absorption spectrometry (Perkin Elmer 3110). Five replicates for each of the four plant species were performed. Blanks and certified reference material were included for procedure control. The difference between concentrations of washed and non-washed samples was considered as the metal concentration on leaf surfaces.

### Chlorophylls (a and b), Carotenoids, and Xanthophyll Concentrations

Pigments from leaves were extracted in acetone (80% v:v) and the concentration was calculated according to the formula proposed by Lichtenthaler and Wellburn (1983). The result was expressed in mg g^-1^ of leaf fresh weight. Five replicates for each composed leaf sample of the four plant species were analyzed.

### Leaf Area

The leaf area is expressed in cm^2^ per gram of fresh leaf using a leaf area integrator LI-COR LI3100C. A representative composed leaves sample from each one of the four plants and conditions (MR and NC) were taken (*n* = 5). It consisted of young and old leaves without visible symptoms of disease or insect activity (Sánchez-López et al., 2015a). In this way, taking the whole specimen was not needed and the plants were preserved in their habitat to allow colonization of MR continues.

### Scanning Electron Microscopy (SEM) Analysis

To observe leaf surface morphology, samples were analyzed under a Scanning Electron Microscope (SEM; JEOL JSM-IT300) at the Microscopy Unit of Biomimic Cluster of National Institute of Ecology. Segments of 1 cm^2^ of previously dehydrated leaf samples were cut out and placed on a sample holder covered with carbon conductive tape. Then, samples were covered with a 15-nm gold layer. Images were taken from both upper and underside of leaves (abaxial and adaxial, respectively), at an accelerating voltage of 10 kV. To verify the presence of PTE-containing particles on leaf surfaces elemental composition of randomly selected particles or leaf areas was analyzed with a SEM coupled with an Energy-Dispersive X-Ray microanalyzer (EDX OXFORD Instruments AzTec). The counting time for point X-Ray spectra was 60 s and 2 min for mapping X-ray; detection limit was 0.1% and 30 kV of accelerating voltage.

### Isolation of Epiphytic Bacteria

For each plant species and condition, leaves with no signs of PTE toxicity, herbivory, or any other physical damage were collected from the fields and, to avoid contamination, immediately kept in sterile containers till processing in the laboratory. Epiphytic microorganisms were isolated through leaf washing technique. Five separated composed samples (each of 250 mg of fresh leaves) of each plant species were washed with 10 mL of a sterile isotonic solution containing 0.01% Tween 80 by shaking for 1 min in a sonic bath at 40 kHz. A 200-μL aliquot of leaf wash solution and of each dilution (10^-1^, 10^-2^, and 10^-3^) were plated on nutrient agar. The plates were incubated for 1 week at 30°C in darkness. Subsequently, colony-forming units (CFU) were counted and calculated per cm^2^ of the leaf. Each morphologically different colony type (size, margin, pattern, opacity, pigmentation, elevation, surface, and consistency) observed on the agar plates set of each of the four plant species was chosen for further functional characterization (see next section). The diversity index was calculated according to the Shannon index (Spellerberg and Fedor, 2003), taking into account morphologically different bacterial colonies.

### Functional Characterization of Isolates

Before the performance of tests described below, bacterial cultures were grown in nutrient broth, then centrifuged (4,000 rpm 10 min) and the cell package washed and resuspended (10^6^ CFU mL^-1^) with sterile distilled water. All tests were performed in triplicate.

#### Tolerance to PTE

The isolates were tested for their PTE tolerance using 1/10 diluted nutrient broth with the addition of 40, 80, 160, and 300 mg L^-1^ of Zn (ZnSO_4_); 45, 90, and 180 mg L^-1^ of Cd (CdSO_4_), 25, 50, and 100 mg L^-1^ of Cu (CuSO_4_); 10, 30, and 60 mg L^-1^ of Sb [K(SbO)C_4_H_4_O_6_], or 80 mg L^-1^ of Pb (Pb(NO_3_)_2_). Fifty microliters of bacterial culture was added to 5 mL of nutrient broth containing PTE.

#### Plant Growth Promotion-Related Traits

Functional characterization of epiphytic bacterial isolates included the production of indole acetic acid (IAA), nitrogen fixation, and phosphate solubilization. The last one was evaluated in National Botanical Research Institute’s phosphate growth solid medium (Nautiyal, 1999), 5 μL of bacterial culture were added on solid medium, after 1-week incubation in darkness the presence of a solubilization halo was considered as a positive result. Nitrogen-fixing capacity was screened in a semi-solid malate-sucrose medium with bromothymol blue as a pH indicator, the same medium supplemented with 0.12 g L^-1^ NH_4_Cl was used as a positive control. An aliquot of 10 μL of washed bacterial inoculum was added to a tube containing 5 mL of the semi-solid medium. After 1 week of darkness incubation, a color change from blue to yellow indicated nitrogenase activity (Croes et al., 2013). Bacterial IAA production capacity was tested in 1/10 nutrient broth after 5 days incubation cultures were centrifuged (3,220 *g*, 15 min), and the supernatant was mixed with Salkowski’s reagent; a pink color was considered positive for IAA production (Glickmann and Dessaux, 1995).

#### Tolerance to UV Radiation

To test tolerance to UV radiation, 100 μL of washed bacterial culture were spread on nutrient agar plates and exposed for 30 min to a UV light germicide lamp (OSRAM G30 T8 30 W). After irradiation, plates were carefully wrapped in aluminum foil and maintained and incubated under dark conditions to avoid exposition to light and thus photo repair processes (Sundin and Jacobs, 1999; Hirsch et al., 2004). Those isolates that were able to grow after the first UV irradiation were exposed to more extended periods (0.5, 1, 3, 6, and 12 h) to UV light.

#### Drought Tolerance

Drought tolerance was tested following the methodology proposed by Hirsch et al. (2004). Plates containing a 2-mm layer of nutrient agar were inoculated and then incubated at room temperature for approximately 4 weeks to complete dryness of the culture medium. Then, bacterial cultures were evaluated for their drought tolerance after 2, 4, 6, and 8 weeks. Controls were inoculated accordingly but incubated in sealed plates. Aliquots of 1 mL of sterile distilled water were placed onto dry bacterial cell material for 15 min, then scraped off, and streaked onto fresh plates. Bacterial growth was verified after 2 weeks of incubation.

### Statistical Analysis

Since each plant species has different leaf morphology and in general leaf characteristics, the statistical comparison of leaf characteristics (CFU, leaf area, and photosynthetic pigments) was made in each species between the two studied conditions (MR and NC). The data were tested for normal distribution and equal variance using the Shapiro–Wilk and the Bartlett test, respectively. Then, a *post hoc* paired Mann–Whitney *U* test (*U*; *p* = 0.05) was applied to compare evaluated variables between the two studied conditions (MR and NC) in each plant species. Correlation analysis was performed according to Spearman’s rank test. To identify the possible influence of factors on the epiphytic bacteria, a principal component analysis (PCA) was applied to the dataset (included variables were as follows: concentration of PTE on leaf surface and inside leaf tissue, CFU, diversity index, leaf area, and concentration of photosynthetic pigments); significant changes were tested using analysis of similarities (ANOSIM). Statistical analysis was performed using PAST software (Hammer et al., 2001).

## Results

### PTE Concentration in the Rhizosphere

The total concentration of PTE in the rhizosphere of collected plants is presented in Supplementary Table S1. In general, the order of PTE abundance was As > Zn > Pb > Cu > Sb > Cd > Ag. The case of As was outstanding, the range of concentrations varied between 5,373 (*B. veronicifolia*) and 21,125 mg kg^-1^ (*Gnaphalium* sp.) in MR rhizospheres (Supplementary Table S1), it means that around the 2% of the rhizosphere of *Gnaphalium* sp. was composed only by As. By contrast, As concentrations in the NC were found between 1,100 and 1,985 mg kg^-1^. *Gnaphalium* sp. rhizosphere in MR also contained the highest concentration of Pb (1,257 mg kg^-1^) and Sb (162 mg kg^-1^). The highest concentrations of Cd (33 mg kg^-1^) and Ag (10 mg kg^-1^) were observed in the rhizosphere *B. veronicifolia*. For the other PTE, the maximum concentrations were 1,075 mg Cu kg^-1^ and 5,114 mg Zn kg^-1^, in *A. choisyi* and *F. trinervia*, respectively.

For the four plant species, the concentration of all PTE was higher in the rhizosphere of plants collected in MR than in the NC. Zinc was the element with the highest differences between conditions being up to 171, 84, 73, and 48× higher in the case of rhizospheres from *Gnaphalium* sp., *F. trinervia*, *B. veronicifolia*, and *A. choisyi*, correspondingly. Antimony rhizospheric concentrations were those with the lowest difference between conditions (around 1×) in the case of *B. veronicifolia*, *A. choisyi*, and *F. trinervia* (Supplementary Table S1). *Gnaphalium* sp. rhizosphere presented the highest difference in PTE concentrations between MR and NC; 80× for Cu, 35× for Pb, 15× for As, and 6× for Sb. In the case of Cd, the most outstanding difference (40×) was observed in *F. trinervia* rhizosphere.

### PTE Concentration on Leaves

**FIGURE 1:**
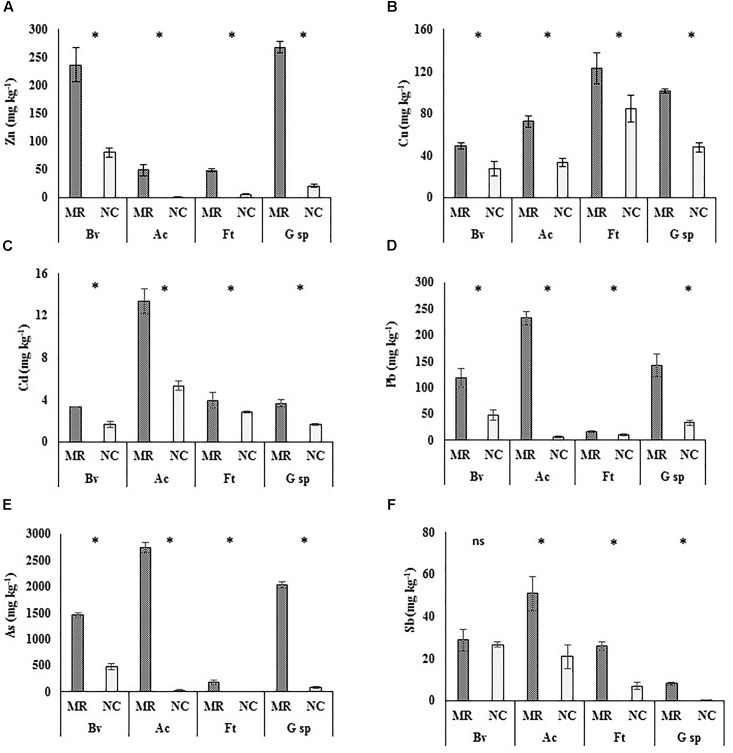
Concentrations of potentially toxic elements on leaf surfaces of four different plant species. Mean ± standard deviation (*n* = 5). Leaves samples collected from mine residues (MR) or non-contaminated control site (NC) of four plant species: *Brickellia veronicifolia* (Bv), *Allionia choisyi* (Ac), *Flaveria trinervia* (Ft), and *Gnaphalium* sp. (G sp.). According to the Mann–Whitney *U* test, (^∗^) means significant and (ns) means non-significant difference (*p* < 0.05). **A** = Zn, **B** = Cu, **C** = Cd, **D** = Pb, **E** = As, **F** = Sb.

The concentration of PTE on leaf surface is shown in Figure 1. In almost all cases, except Sb in *B. veronicifolia* (Figure 1F), the concentration of PTE was significantly higher in samples from MR than in those from the NC. *A. choisyi* was interesting since its leaves when growing on MR, had 83× higher concentrations of As, 66× of Zn, 35× of Pb, and 3× of Cd compared to NC leaves. Regarding As (Figure 1E), besides *A. choisyi*, *F. trinervia* (34×), and *Gnaphalium* sp. (22×) also presented higher concentrations on leaves from MR samples than those from the control ones. The case of *Gnaphalium* sp. was outstanding since it retained 16× more Sb on MR leaves than in those from the NC. For Cu, the difference in metal concentrations on leaves was not as high as As, Zn, Pb, and Sb; however, both *A. choisyi* and *Gnaphalium* sp. retained in their leaves twice the concentration of Cu when growing on PTE-containing MR than those from NC (Figure 1B).

Regarding MR samples, *A. choisyi* had outstanding concentrations of Pb (232 mg kg^-1^), Cd (13 mg kg^-1^), As (2,728 mg kg^-1^), and Sb (51 mg kg^-1^), which were above concentrations of the other species (Figures 1D,C,E,F, respectively). *Gnaphalium* sp. (281 mg kg^-1^) and *B. veronicifolia* (237 mg kg^-1^) presented the highest concentration of Zn (Figure 1A), *F. trinervia* and *Gnaphalium* sp. were outstanding plant species for the accumulation of Cu on leaf surfaces, with 128 and 110 mg of Cu kg^-1^ (Figure 1B). In the case of samples collected in NC, high concentrations of PTE were observed on leaves of *B. veronicifolia* (80 mg Zn kg^-1^, 48 mg Pb kg^-1^, 470 mg As kg^-1^, 27 mg Sb kg^-1^), *F. trinervia* (85 mg Cu kg^-1^), and *A. choisyi* (5 mg Cd kg^-1^).

### Plant Parameters

The evaluated variables related to plant characteristics, including leaf area and concentrations of chlorophylls, xanthophylls, and carotenoids were variable according to pollution conditions and plants species (Table 1). The leaf area was significantly different between states; this was only higher in leaves of *A. choisyi* and *F. trinervia* from NC than samples from MR. However, no differences were detected for *B. veronicifolia* and *Gnaphalium* sp. (Table 1). In general, *B. veronicifolia* leaves had higher area per fresh weight (32.9 cm^2^ g^-1^ in MR and 40.2 cm^2^ g^-1^ in NC) compared to the other plant species.

**Table 1:** Leaf area, chlorophylls concentration, colony-forming units, and diversity index of leaves and isolated epiphytic bacteria.

Plant species		Leaf area (cm^2^ g^-1^)	Chlorophyll (μg cm^-2^)			Xanthophylls/carotenoids (μg cm^-2^)	CFU cm^-2^ of fresh leaves	Shannon index
			a	b	total			
*B. veronicifolia*	MR	32.9^ns^	3.8^∗^	0.8^ns^	4.7^∗^	1.2^∗^	1.5 × 10^3∗^	0.190 ^ns^
	NC	40.2	8.3	1.9	10.6	3.0	0.9 × 10^3^	0.175
*A. choisyi*	MR	6.9^∗^	6.7^∗^	1.4^ns^	8.3^∗^	2.0^∗^	32.6 × 10^3∗^	0.505 ^ns^
	NC	11.6	3.7	1.1	5.0	1.2	7.8 × 10^3^	0.466
*F. trinervia*	MR	4.1^∗^	3.3^∗^	0.4^∗^	3.8^∗^	1.0^ns^	16.2 × 10^3ns^	0.334^∗^
	NC	21.5	4.6	1.0	5.8	1.2	13.9 × 10^3^	0.120
*Gnaphalium* sp.	MR	10.5^ns^	5.3^∗^	4.2^∗^	9.9^ns^	0.6^∗^	8.2 × 10^3∗^	0.141^∗^
	NC	12.3	7.9	1.2	9.4	1.8	1.7 × 10^3^	0.267


*MR, mine residues; NC, non-contaminated control sites; CFU, colony-forming units; significant (^∗^) or non-significant (ns) difference between samples of mine residues and control sites in each plant species according to *U* test (*p* < 0.05).*

Significant differences of concentrations of chlorophyll *a*, chlorophyll *b*, and total chlorophyll were found in leaves of *F. trinervia*, the concentrations of the pigments mentioned above were higher in NC than in MR (Table 1). Differences in concentrations of chlorophyll *a* were detected also in *B. veronicifolia*, *Gnaphalium* sp., and *A. choisyi*. While *A. choisyi* had a higher value at MR (6.7 μg cm^-2^) than in NC (3.7 μg cm^-2^); the opposite result was observed in the other two plant species (Table 1). For chlorophyll *b*, no differences between conditions were observed in *B. veronicifolia* and *A. choisyi*. The concentrations of the mentioned pigment were significantly different for *F. trinervia* and *Gnaphalium* sp., being NC samples from *F. trinervia* those with higher concentration (1.0 μg cm^-2^) than this in MR samples (0.4 μg cm^-2^). By contrast, in *Gnaphalium* sp., the values of chlorophyll *b* were higher in MR (4.2 μg cm^-2^) samples than in control ones (1.2 μg cm^-2^) (Table 1). Regarding the total concentration of chlorophyll, significant differences were detected in leaves from all species except *Gnaphalium* sp. Both *B. veronicifolia* and *F. trinervia* had a high concentration of total chlorophyll in NC; contrary to *A. choisyi* where leaves from MR had the higher concentration than leaves from the NC (Table 1).

The concentration of xanthophyll and carotenoids was different in leaves samples of *B. veronicifolia, Gnaphalium* sp., and *A. choisyi* from MR or NC samples (Table 1). Low concentrations were observed in leaves from *F. trinervia* and *Gnaphalium* sp. from MR plants (1.0 and 0.6 μg cm^-2^, respectively). The opposite trend was observed in leaves of *A. choisyi* (8.3 μg cm^-2^ from MR plants and 5.0 μg cm^-2^ from NC samples).

### Scanning Electron Microscopy (SEM) Analysis

**FIGURE 2:**
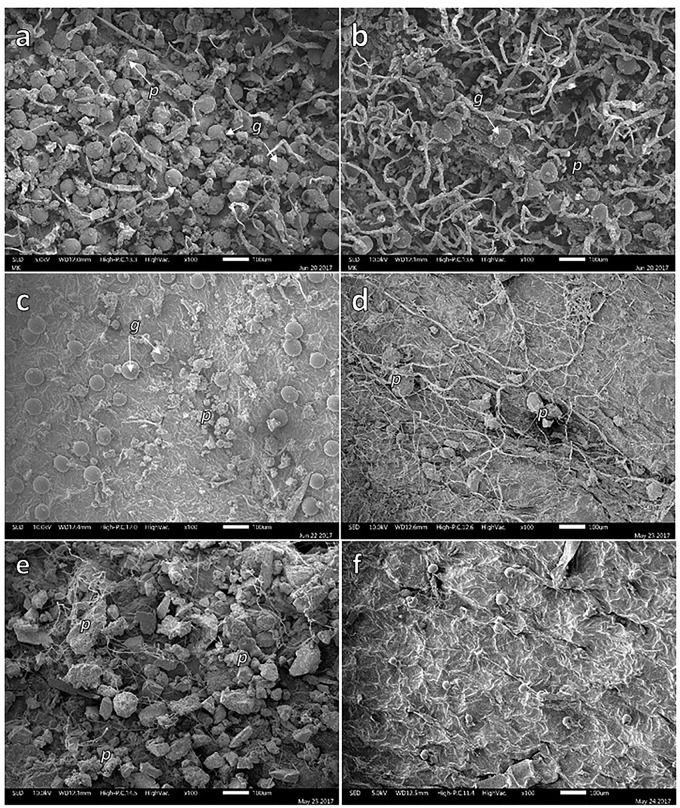
SEM images of solid particles on the adaxial **(a,c,d,f)** and abaxial face **(b,e)** of leaves of *Brickellia veronicifolia* **(a–c)** and *Allionia choisyi* **(d–f)** collected from mine residues **(a,b,d,e)** or non-polluted control site **(c,f)**. Particles = *p*; glands = *g*.

Particles were present on adaxial and abaxial leaf surface of the four studied plant species. Figure 2 shows the presence of particles on the adaxial and abaxial surface of *B. veronicifolia* (Figures 2a,b) and *A. choisyi* (Figures 2d,e). Particles were present in leaf samples collected from MR and also from the NC. Nevertheless, the presence of particles was more evident in the case of MR leaves than these from NC (Figure 2); for example, the adaxial surface of *B. veronicifolia* leaves from MR (Figure 2a) and NC (Figure 2c). Similarly, particles are more abundant in *A. choisyi* adaxial surface in MR (Figure 2d) than in NC leaves (Figure 2f).

**FIGURE 3:**
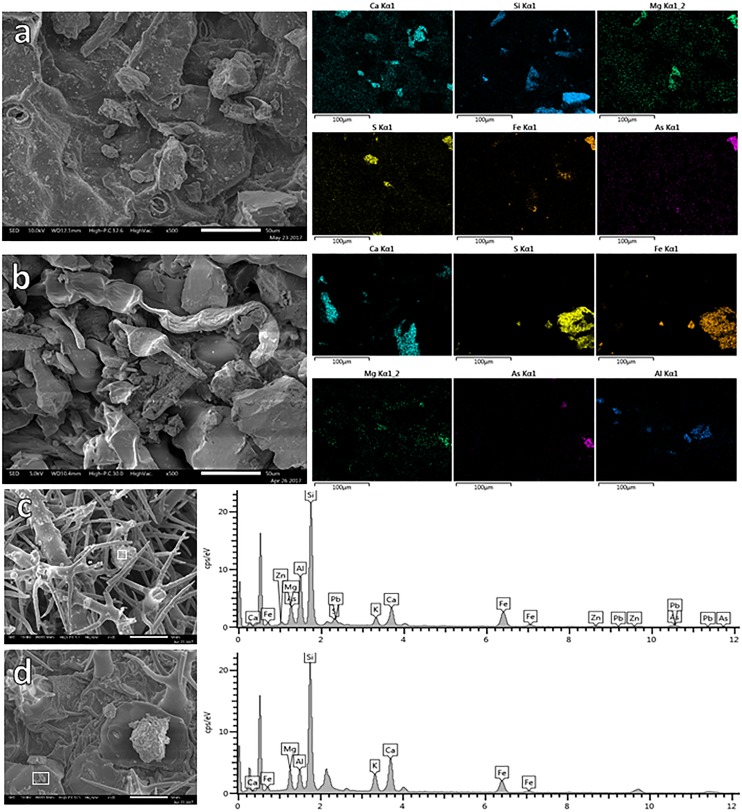
EDX-mapping of leaf surface of *Flaveria trinervia* collected on control site **(a)** and mine residues **(b)**. EDX-spectrum of specific particles (white square) retained on leaves of *Gnaphalium* sp. growing on mine residues **(c)** or in the non-contaminated control site **(d)**.

Through the application of X-ray mapping, it was possible to identify the presence and distribution of PTE in specific areas of the leaf surfaces (Figure 3). Certain elements such Ca, Si, and Mg are widely spread on the adaxial leaf surface of *F. trinervia* from NC, while on leaves of the same plant species from MR other elements (S, Fe, for instance) were more common; PTE like As were detected too (Figure 3b). The same trend was observed for the other studied plant species. The use of accurate X-ray analysis allowed distinguishing the composition of specific particles. Most of the particles were composed by elements like Ca, K, Si, and Fe; these elements were commonly detected on samples from MR and NC as shown for *Gnaphalium* sp. (Figures 3c,d). However, other elements considered as PTE (Cu, Zn, As, Pb, and Sb) were only observed in MR leaves (Figure 3c), but not on leaves collected at the NC (Figure 3d).

**FIGURE 4:**
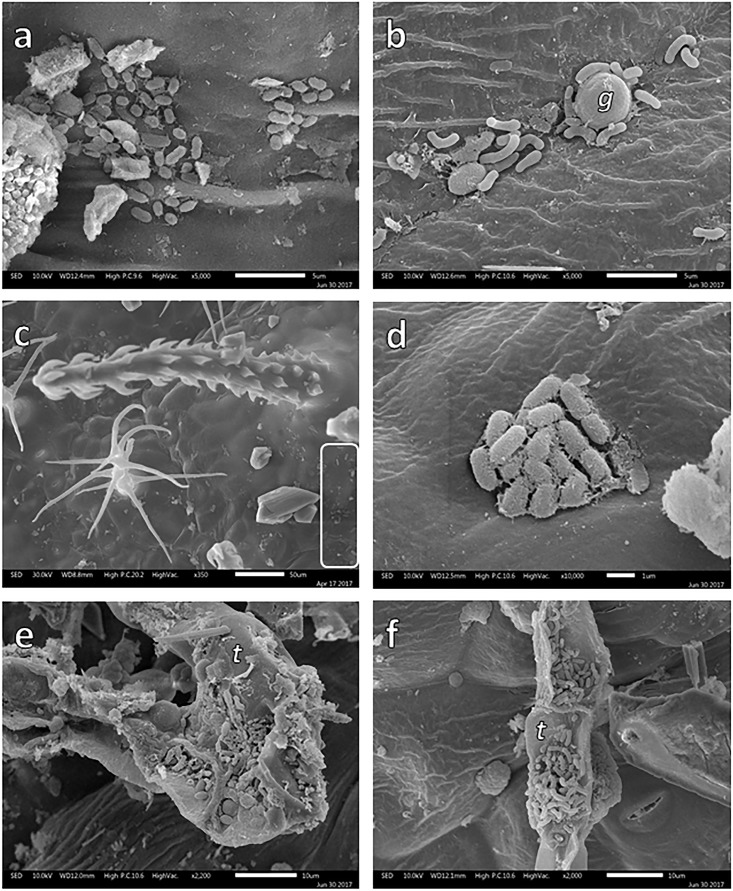
SEM images of bacterial colonies growing on the leaf surface of *Brickellia veronicifolia* **(a,b)**, *Gnaphalium* sp. **(c,d)**, and *Flaveria trinervia* **(e,f)** colonizing mine residues. Glands = *g* and trichomes = *t*.

During SEM analysis, some bacterial colonies were observed on leaf surfaces (Figure 4). In the case of *B. veronicifolia* leaves, colonies were detected in gullies (Figure 4a) and in the nooks formed between glands and leaf surface (Figure 4b). In *Gnaphalium* sp., epiphytic bacteria were spotted on the leaf surface under trichomes and in crevices (Figures 4c,d). For *F. trinervia*, abundant bacteria colonies were frequently observed in crevices located on trichomes surfaces (Figures 4e,f).

### Bacterial Isolates

The number of CFU was variable among species in each Condition; except for leaves of *F. trinervia*, significant higher amount of CFU from leaves of all plants from MR *vs* these from NC were observed (Table 1). Leaves of *Gnaphalium* sp. and *A. choisyi* from MR had 5× and 4× more CFU, respectively, than those from the NC. The highest amount of CFU per cm^2^ was observed in *A. choisyi* leaves collected in MR (32.6 × 10^3^), while leaves of *B. veronicifolia* from the NC had the lowest value (0.9 × 10^3^).

The morphological diversity of epiphytic bacterial isolates measured with Shannon index was variable (Table 1). However, no significant differences between polluted and control condition were detected in leaves of *B. veronicifolia* or *A. choisyi*, the diversity index of *Gnaphalium* sp. and *F. trinervia* leaves differed between pollution conditions. However, these two plant species had different trends. In the case of *Gnaphalium* sp. leaves, the diversity of epiphytic isolates was higher in the NC (0.267) than those in MR (0.141). The opposite was observed in *F. trinervia* leaves, where the diversity index was greater in samples from PTE-containing MR (0.334) than those from the NC (0.120).

### Functional Characterization of Isolates

#### Tolerance to PTE

Potentially toxic element-tolerant epiphytic bacteria were isolated from leaves of plants growing on MR and the NC. However, tolerance to PTE was observed more frequently in isolates from MR samples than in those from the NC (Table 2). In the case of epiphytic bacteria isolated from *B. veronicifolia* leaves, all isolates from MR were able to grow in the presence of 40 mg Zn L^-1^, and four isolates out of 10 were tolerant to the highest tested concentration of Zn (160 mg L^-1^). By contrast, only one of the strains from the same plant species but isolated from the NC grew in medium containing 160 mg L^-1^ (Table 2). Two bacterial isolates out of 10 from MR showed to be tolerant to 150 mg of Pb L^-1^; by contrast, none from the NC grew at such concentration (Table 2). No *B. veronicifolia* isolate, neither from MR nor NC tolerated the highest tested concentrations of Cu (50 mg Cu L^-1^), Cd (90 mg L^-1^), or Sb (60 mg L^-1^). Nevertheless, seven, one, and six out of 10 epiphytic isolates from MR leaves grew in medium containing the lowest concentrations of Cu (25 mg L^-1^), Cd (45 mg L^-1^), and Sb (10 mg L^-1^), correspondingly (Table 2). By contrast, only the two, one, and two out of seven NC bacterial isolates tolerated the concentrations mentioned above Cu, Cd, and Sb, correspondingly (Table 2). Among the more outstanding epiphytic bacterial isolates are BA6 and BA15, both isolated of *B. veronicifolia* leaves from MR. These two strains grew in medium containing 150 mg Zn L^-1^, 30 mg Sb L^-1^, or 25 mg Cu L^-1^. Strain BA6 tolerated up to 150 mg Pb L^-1^ and BA15 up to 80 mg Pb L^-1^ (Supplementary Table S2).

**Table 2:** Number of epiphytic bacterial isolates tolerant to potentially toxic elements.

Plant species		# of total isolates	Zn (mg L^-1^)			Cu (mg L^-1^)		Cd (mg L^-1^)		Pb (mg L^-1^)		Sb (mg L^-1^)		
			40	80	160	25	50	45	90	80	150	10	30	60
*B. veronicifolia*	MR	10	10	6	4	7	0	1	0	9	2	6	5	0
	NC	7	5	1	1	2	0	1	0	2	0	2	1	0
*A. choisyi*	MR	9	8	5	3	6	3	2	0	7	2	7	4	0
	NC	8	4	4	3	3	0	2	0	3	0	8	4	0
*F. trinervia*	MR	12	9	5	1	8	0	4	0	7	1	5	2	0
	NC	8	4	3	2	3	0	2	0	5	0	6	1	0
*Gnaphalium* sp.	MR	10	9	3	1	2	1	6	2	6	2	5	2	0
	NC	3	2	1	1	1	0	1	0	1	1	1	1	1


*MR, mine residues; NC, non-contaminated control sites; # total isolates, number of characterized isolates.*

In the case of phylloplane isolates of *A. choisyi*, it was interesting to notice that all strains (eight) from the NC site were tolerant to 10 mg Sb L^-1^, in contrast only seven out of nine from the MR (Table 2). As observed in *B. veronicifolia* isolates, no *A. choisyi* strain isolated from leaves from the control site was able to grow in medium containing the highest concentrations of Cu (50 mg L^-1^), Cd (90 mg L^-1^), or Pb (150 mg L^-1^). MR isolates were not tolerant of the highest Cd concentration tested (90 mg L^-1^). However, three and two out eight epiphytic bacteria of *A. choisyi* leaves collected from MR were able to grow in the highest concentrations of Cu and Pb, respectively (Table 2). Among *A. choisyi* epiphytic isolates DA15 showed tolerance to 150 mg Zn L^-1^, 50 mg Cu L^-1^, 45 mg Cd L^-1^, 80 mg Pb L^-1^, and 30 mg Sb L^-1^ (Supplementary Table S2).

Epiphytic bacterial isolates of *F. trinervia* showed tolerance to Zn; however, contrary to the general trend, strains tolerant to the highest Zn concentration (160 mg L^-1^) were more frequent in control samples (two bacteria out of eight) than in MR ones (one out of 12). By contrast, tolerance to the highest Pb tested concentration (150 mg L^-1^) was observed only in one out of 12 MR isolates (Table 2). No *F. trinervia* isolate either from MR or NC was able to grow in medium supplemented with 50 mg Cu L^-1^, 90 mg Cd L^-1^, or 60 mg Sb L^-1^ (Table 2). About *Gnaphalium* sp. epiphytic bacteria, the only strain tolerant to 60 mg L^-1^ of Sb, the highest tested concentration, was GN3 isolated from the NC (Table 2). GA4 and GA6 isolates from MR *Gnaphalium* sp. (Supplementary Table S2) were the only strains that tolerated up to 90 mg L^-1^ of Cd.

#### Plant Growth Promotion-Related Traits

Functional traits related to plant growth promotion (IAA production, nitrogen fixation, and phosphate solubilization) and plant protection (tolerance to drought and UV radiation) were variable among plant species and between conditions (Table 3). Concerning *B. veronicifolia* leaves, production of IAA was observed more often in the NC isolates (two out of seven isolates) than in those from MR (one out of 10 isolates). The same trend was observed for nitrogen fixation, where one out 10 isolates of MR epiphytes and three out of seven of NC isolates possessed such trait (Table 3). The proportion of occurrence of phosphate solubilization was similar between conditions (Table 3).

**Table 3:** Plant growth promotion and plant protection related traits of isolated epiphytic bacteria.

Plant species		# of total isolates	IAA production	N_2_ fixation	PO_4_ solubilization	UV tolerance (h)^§^	Drought tolerance (weeks)^§^
*B. veronicifolia*	MR	10	1	1	3	4.2	4.2
	NC	7	2	3	2	2.3	2.4
*A. choisyi*	MR	9	2	1	3	3.3	2.7
	NC	8	1	0	2	2.1	3.0
*F. trinervia*	MR	12	2	3	6	0.5	3.9
	NC	8	1	3	2	3.5	3.0
*Gnaphalium* sp.	MR	10	1	1	3	3.5	3.4
	NC	3	2	0	1	1.2	1.3


*MR, mine residues; NC, non-contaminated control sites; # of total isolates, number of characterized isolates; IAA, indole acetic acid; ^§^ average of UV light or drought exposition taking into account the number of isolates of each plant species in each condition.*

Regarding *A. choisyi* epiphytes, the production of IAA was more common to detect in MR (two out of nine) isolates than in those from the NC (one out of eight bacteria, Table 3). Nitrogen fixation and phosphate solubilization presented a similar trend; higher number of strains isolated from MR than CS. In the first case, one out of nine isolates had positive results, but none with such characteristic was observed among NC isolates. One out of nine of the cultivable epiphytic bacteria MR *A. choisyi* was capable of solubilizing phosphate, compared to the two out of eight of these from NC isolates (Table 3).

Regarding the epiphytic bacteria of *F. trinervia*, it was outstanding that the half and a quarter of the 12 epiphytic isolates from MR leaves were able to solubilize phosphate and to fix nitrogen, respectively. These values were higher than those observed in the same plant species but collected in the NC (Table 3). Among all four different plant species growing on MR, these from *F. trinervia* had the highest proportions of bacteria positive for nitrogen fixation and phosphate solubilization. In the case of *Gnaphalium* sp. leaves, none of the NC isolates was positive for nitrogen fixation test (Table 3). The number of bacterial epiphytes capable of phosphate solubilization was similar when isolated from MR (one out of 10) and the NC (one out of three) samples, whereas IAA production appeared to be most common in NC isolates (two out of three) than in those from MR (one out of 10, Table 3).

Among all epiphytic isolates only three strains presented these three characteristics together (IAA production, nitrogen fixation, and phosphate solubilization): BN3, DA16, and FA13 (Supplementary Table S3). The last two were isolated from leaves of *A. choisyi* and *F. trinervia*, respectively, both from MR. BN3 was detected on control *B. veronicifolia* leaves.

#### UV and Drought Tolerance

For *B. veronicifolia* epiphytic isolates, the average exposition time to drought (4.2 weeks) and UV radiation (4.2 h) was longer in strains from MR leaves than in strains of the NC (2.4 weeks and 2.3 h, respectively). The average UV radiation (4.2 h) and drought (4.2 weeks) exposition in *B. veronicifolia* epiphytes in MR were the highest observed (Table 3). In similarity with these observations, for *A. choisyi*, the average UV exposure for epiphytes isolated from MR leaves was higher (3.3 h) than in those from the NC (2.1 h). The drought tolerance was 2.7 and 3.0 weeks for MR and NC strains, correspondingly (Table 3). The same trend, high tolerance in MR epiphytes was detected in *Gnaphalium* sp. For UV and drought tolerance, the results (Table 3) showed that these traits were more common in epiphytic bacteria from the PTE polluted site (3.5 h in UV, 3.4 weeks drought) than these from the NC (1.2 h in UV, 1.3 weeks drought).

The epiphytic isolates from *F. trinervia* in MR presented low tolerance to UV (30 min), compared to those of the same plant species but from the NC (3.5 h). The average time exposition to UV was seven times higher for epiphytes from the NC (Table 3). The opposite was observed for drought tolerance, MR epiphytes had an average tolerance of 3.9 weeks and 3 weeks for the NC ones.

Three different epiphytic isolates exhibited tolerance to the maximum levels of UV (12 h) and drought (8 weeks) tested in this research: BA15, DN7, and FN9; isolated from leaves of *B. veronicifolia* in MR, and from *A. choisyi* and *F. trinervia* of NC, respectively (Supplementary Table S3).

### Differences and Similarities of Epiphytic Bacteria Cultivable Community

**FIGURE 5:**
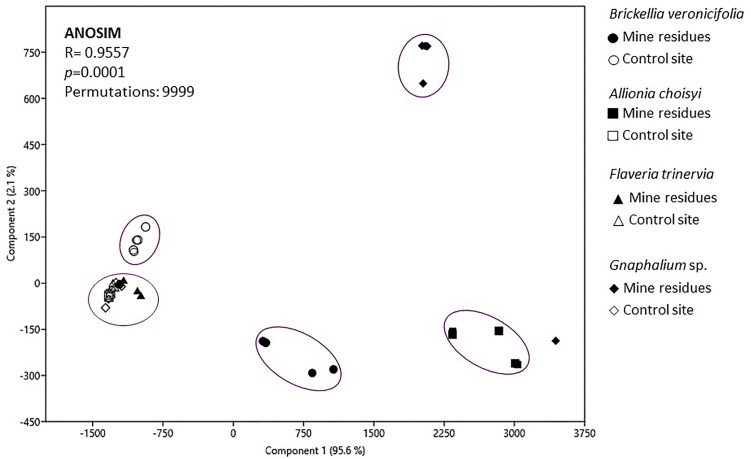
Principal component analysis of variables characterizing leaf surfaces of four different plant species growing on mine residues (MR) or non-contaminated control site (NC). ANOSIM, analysis of similarities.

To compare plant species and conditions, a PCA was applied. The resulting plot showed that 97.7% of the variance was explained by the two first components and grouping of samples was observed too (Figure 5). In general five groups were identified; the first one included three out of the four analyzed plant species: *Gnaphalium* sp., *A. choisyi*, and *F. trinervia*, these three from the NC. The other four groups were formed by only one plant species in one condition, *B. veronicifolia* from the control site is a second group. *Gnaphalium* sp., *B. veronicifolia*, and *A. choisyi*, all three of MR, formed each of them a separate group. According to ANOSIM, this grouping was significant (Figure 5). These results showed that the leaf characteristics of *B. veronicifolia, Gnaphalium* sp., and *A. choisyi* are different when coming from MR or NC. Moreover, leaf characteristics significantly differed among plant species when were found on PTE containing MR, except for *F. trinervia*. By contrast, when plants were collected from the respective NC, the leaf characteristics were similar among them, excluding *B. veronicifolia*.

Complementary, correlation analysis demonstrated (Supplementary Table S4) that the number of CFU of epiphytic bacteria was positively affected by the concentration of Cu, Cd, Sb, Pb, and As on non-washed leaves. By contrast, the diversity of epiphytic bacteria, estimated by Shannon index, was negatively influenced by the concentration of Pb and As in leaf tissue (Supplementary Table S4).

## Discussion

A study of epiphytic bacteria isolated from four different plant species suitable for use as phytobarriers was carried out. It was demonstrated that the four studied plant species (*B. veronicifolia*, *A. choisyi*, *F. trinervia*, and *Gnaphalium* sp.) retained considerable amounts of PTE-containing particles on their leaf surfaces (Figures 1, 3). Therefore, the isolated epiphytic bacteria coexist on the phylloplane with important concentrations of diverse PTE, such as Cd, Zn, Pb, Cu, As, and Sb. Moreover, since the study site is located in a semi-arid area, epiphytes can cope with restrictiave environmental conditions including extreme temperatures, rain scarcity, and high UV radiation, among others.

*Allionia choisyi* was the most outstanding plant species regarding the concentration of Cd, Sb, As, and Pb on its leaves surfaces followed by *Gnaphalium* sp. (Cu, Zn, and As) and *B. veronicifolia* (Zn and As) (Figure 1). *B. veronicifolia, F. trinervia*, and *Gnaphalium* sp. were previously reported for their capacity to retain different PTE on their surfaces (Sánchez-López et al., 2015a). However, this is the first report of *A. choisyi* as a potential phytobarrier.

In the present research, *B. veronicifolia* followed by *Gnaphalium* sp. presented high capacity of PTE retention on leaves; this is estimated as the quotient between the concentrations of certain PTE in the non-washed sample divided by the concentration in the washed sample. Following this observation, the most outstanding epiphytic bacteria regarding PTE-tolerance were isolated from *B. veronicifolia* (BA6 and BA15) and *Gnaphalium* sp. (GA4, GA6, Supplementary Table S2) of MR. It is documented that different plant-associated bacteria including rhizobacteria and endophytes of varying plant organs can be equipped with a PTE-resistance/sequestration system able to decrease PTE phytotoxicity or enhance their accumulation in plant tissues (Weyens et al., 2009; Rajkumar et al., 2012; Ma et al., 2016). The same behavior might be expected for phylloplane bacteria (Weyens et al., 2015). To the best of our knowledge, there is no available information regarding PTE tolerance of epiphytic bacteria isolated from leaves to which we can compare our results. Nevertheless, taking into account previous report about seed endophytic bacteria of *Crotalaria pumila* growing in the same MR (Sánchez-López, 2015), epiphytic bacteria tolerated lower concentrations of Zn, Cd, and Cu than endophytic bacteria. The highest concentrations, in mg L^-1^, in which epiphytic bacteria grew were 150 of Zn, 90 of Cd, and 50 of Cu (Table 2), although endophytes tolerated up to the double concentration of the mentioned elements (Sánchez-López, 2015). However, from the results of the present research, it has been noted that some strains from MR were able to tolerate the highest PTE concentrations tested. Epiphytic strains isolated from NC did not grow in the highest concentrations of Cu and Cd, but they did in the lowest ones (Table 2). This can be explained by the higher prevalence of diverse PTE in the MR were plants were sampled (Supplementary Table S1) as reported by Baldi et al. (1995). This information suggests that epiphytic bacteria isolated from MR possess PTE detoxification mechanisms that allow them to may help their host plant to cope with PTE and the stresses caused by such elements. An exception was observed in NC strains from *Gnaphalium* sp. leaves, which tolerated the highest Pb concentration and were the only ones that grew at the highest Sb concentration. Hence, the role of microbes in the tolerance to PTE on the surface of leaves has to be yet studied in more detail.

The obtained information showed the epiphytic bacterial colonies found on the leaves of *B. veronicifolia*, *F. trinervia*, *A. choisyi*, and *Gnaphalium* sp. inhabited in the presence of high concentrations of different PTE. SEM analysis demonstrated the presence of bacterial colonies on different structures of leaf surfaces, mainly on the tip of trichomes, but also underneath glands, and crevices between epidermal cells (Figure 4). Separate leaf appendixes, trichomes, and crevices principally, present an increased nutrient availability that can be used for bacterial cells (Leveau and Lindow, 2001); therefore, these can be expected locations for epiphytic bacteria colonization (Monier and Lindow, 2004; Remus-Emsermann et al., 2014). During sampling, only healthy leaves were taken; too old or too young leaves, necrotic or yellowish, damaged leaves were not sampled. Then, the epiphytic bacteria observed on leaf samples were not causing visible damage or disease to their hosting plant. These characteristics (no causing damage, ability to grow in the presence of PTE) plus the plant growth and plant protection traits that the studied epiphytic bacteria exhibited make them of particular interest for air phytoremediation purposes.

In the case of plant growth promotion related traits (production of IAA, phosphate solubilization, and nitrogen fixation), nitrogen fixation seemed to be relevant for epiphytic bacteria of *A. choisyi*, *Gnaphalium* sp., and *F. trinervia* when growing on MR (Table 3). The concentrations of nutrients like nitrogen and phosphorous are low in the studied mine deposits (Sánchez-López et al., 2015b); thus, the associated epiphytic microorganisms can provide nutrients to their host plant by the fixation of atmospheric nitrogen in plant leaves (Fürnkranz et al., 2008; Rico et al., 2014). Similarly, phosphate solubilization was an essential characteristic for *F. trinervia* epiphytes when found in MR. Commonly, this trait is determined when analyzing plant–bacteria interactions to select bacteria with plant growth promotion characteristics. Bacteria that are able of P solubilization can provide their host plant with the mentioned nutrient. Moreover, it is known that in the soil, inorganic P reacts with metals and produce insoluble precipitates decreasing availability and risk of these contaminants (Bolan et al., 2003). Hence, P solubilized by bacteria in the phyllosphere may act similarly. However, very novel information shows that phosphate solubilizing bacteria with high gluconic acid production may also dissolve CdCO_3_ and solid Cd from soil (Yang et al., 2018). Hence, future research should be followed to explain much better the repercussion of P solubilized by bacteria in polluted sites and take into account other bacterial biochemical traits related to remediation at the phyllosphere level.

The participation of bacteria into phylloremediation strategy in metal-polluted environments should be understood. It is expected that phyllospheric bacteria offer to their host plant benefits comparable to those described for plant growth-promoting bacteria (PGPB), which is increase in growth and biomass production in plants. Several benefits have been referred to seed isolated bacteria, which may also be suggested for phyllospheric bacteria. For example, higher host’s tolerance to metals (Mastretta et al., 2009; Weyens et al., 2009) and adaptation process of plants for the establishment in a restrictive metal-polluted environments (Truyens et al., 2013, 2016), as well as improvement of plant nutrition, reduction of stress response, transformation of metal, etc. Burch et al. (2016) observed high-level culturability of epiphytic bacteria and high frequency of biosurfactant producers on leaves; which can also be worthwhile to modify metal bioavailability. Future research should explore bacterial gene profiling or gene expression with metagenomic and metatranscriptomics approaches to elucidate about the direct bacterial roles and investigate interactions between leaf surfaces and the dynamics of these communities along the plant life cycle. Therefore, several hypotheses may be tested, for example, the cultivable bacteria isolated from phyllosphere sequester heavy metals at the leaf surface and decrease metal availability; these cultivable bacteria influence metal speciation at the phyllosphere level; these cultivable bacteria have an orchestrated set of mechanisms to help their plant host to deal under heavy metal contamination.

It is mentioned that tolerance to UV radiation is a common feature for leaf-associated bacteria (Sundin and Jacobs, 1999). In our research, this characteristic was more common to detect in epiphytic bacteria isolated from MR leaf samples; these isolates tolerated longer exposition to UV light and drought than those from the NC. In some cases, the difference was more than the double of time (Table 3). The same behavior was observed for drought stress, where epiphytic bacteria of MR plants were able to cope with long periods of drought than bacteria of NC plants.

High tolerance to different PTE, nitrogen fixation, phosphate solubilization, and protection against UV light and drought stress represent, if not essential, highly advantageous traits for plant–epiphytic bacteria association in a semi-arid climate. These are fundamental characteristics for the establishment of a vegetated cover that works as phytobarrier. In other words, epiphytic bacteria can help the establishment of a plant cover that diminishes the aerial dispersion of PTE-associated particles. Epiphytic bacteria can participate in the phylloremediation of air pollutants either, directly acting on air pollutants of indirectly, through plant-growth and plant-protection traits (Weyens et al., 2015; Wei et al., 2017). These traits include those studied in the present research, production of a substance that promotes plant growth (IAA), acquisition of nutrients (N fixation, phosphate solubilization), and tolerance to drought and UV radiation.

Interestingly, the most outstanding bacterial strains regarding tolerance to PTE, UV, and drought resistance were observed in *B. veronicifolia*, a plant species previously reported as a candidate for phytobarrier (Sánchez-López et al., 2015a) and found in considerable amounts spontaneously colonizing PTE containing MR (Sánchez-López et al., 2015b). Of particular interest is isolate BA15, which is a candidate to conduct further experiments to elucidate its possible beneficial effects for the phytoremediation of air pollutants. Our research group is performing these experiments. The identification of the aforementioned and other potential epiphytic isolates is being currently carried out by our research group. It is likely that the noteworthy epiphytic bacteria of the studied plant species also participate in the phytoremediation of other air pollutants as observed by other authors for volatile organic compounds or other inorganic compounds such as SOx and NOx (Weyens et al., 2015; Wei et al., 2017). This topic will be investigated in the future.

Additionally, more functional traits of the epiphytic bacterial isolates can be yet explored, for instance, the production of compounds with surfactant properties (Burch et al., 2016; Thapa et al., 2017). Since epiphytic bacteria can act as a barrier against plant pathogens (Perazzolli et al., 2014) beneficial epiphytic communities could be seen as potential plant probiotic agents (Innerebner et al., 2011). The exploration of the biocontrol potential of the epiphytic isolates is suggested as well. It is also important to note that in this research we took into account only bacteria, for future research, it is recommended to investigate the epiphytic fungi too simultaneously.

Even though the phylloplane cultivable bacteria showed to be tolerant to PTE, there were some interesting trends in regard to these elements can affect the quantity and diversity of epiphytes. The cultivable epiphytic community was more abundant in samples collected from MR than these from the NC (Table 1). Dobroné-Toth et al. (2009) observed the same trend in leaves of ragweed (*Ambrosia eliator*) exposed to Zn, Ni, and Cd. Nevertheless, this finding, higher cultivable population of epiphytes in PTE polluted sites, is contrary to the observations of Brighigna et al. (2000), Joshi (2008), and Khanna (1986). These authors reported a detrimental effect to a potent strong inhibition on the counts of cultivable phylloplane bacterial community due to PTE polluted environment. Joshi (2008) mentioned that the epiphytic bacterial community in the contaminated site was just the half of the counts of the non-polluted site, while Brighigna et al. (2000) found a decrease of 35 times. Joshi (2008) observed a negative correlation between the epiphytic bacterial population of *Alnus nepalensis* and the concentration of Pb, Zn, Cu, and Cd in non-washed samples. Brighigna et al. (2000) attributed this diminish to the quantities of PTE present in the leaf tissue. Our results showed the opposite behavior, the number of CFU was positively correlated to the concentration of Cu, Pb, As, Sb, and Cd in non-washed leaf samples and on the leaf surface (Supplementary Table S4). This can be explained by a possible stimulatory effect of pollutants on the epiphytes counts reported by Manning (1971) who suggested that in a harsh environment the production of propagules ensure the survival or movement of microorganisms to a suitable environment. It has to be taken into account that the previous reports studying cultivable epiphytic bacteria used different isolation protocols; moreover, even if all authors applied the same protocol, the studies were made on different plant species and in urban environments, where besides the pollution by PTE there exist additional emitted pollutants, e.g., NOx, SOx, and volatile organic compounds (Arnfield, 2003; Smets et al., 2016).

Low diversity is also reported as an effect of PTE polluted environments in epiphytic communities (Khanna, 1986; Smets et al., 2016). The results obtained in the present work regarding Shannon morphological diversity index were variable depending on the plant species (Table 1). In agreement with findings of earlier researchers (Brighigna et al., 2000; Joshi, 2008), higher bacterial diversity was detected in the polluted site but only for *F. trinervia* leaves. In *Gnaphalium* sp., the variety of bacterial epiphytes was higher in samples from the NC than these from MR (Table 1). However, the correlation analysis for overall data set demonstrated that the diversity index was negatively correlated to the concentration of Pb and As within leaf tissues, and to the content of total chlorophyll and chlorophyll *b* as well (Supplementary Table S4). It seems that the concentration of As and Pb inside leaf tissues, not on the surface, cause a reduction in the diversity of epiphytic cultivable bacteria. PTE stress can deteriorate the physiological status of the plant (Küpper et al., 2002; Nagajyoti et al., 2010). A diminished physiological activity and productivity of the host plant can cause a decrease in the richness of the epiphytic community (Peñuelas et al., 2012), as a possible consequence of lack of water, nutrients, and organic matter that the plant can supply to their associated microorganisms.

The PCA analysis (Figure 5) showed two main trends: (i) differences between leaves collected from MR and the NC and (ii) when found in a contaminated environment, each plant species is different from the rest. Formerly, Knief et al. (2010) and Finkel et al. (2011) observed that the site-specific environmental factors are significant on the phyllospheric community, in this case, pollution by PTE. However, our results confirmed that besides the presence of pollutants (Rajkumar et al., 2012, 2013; Ma et al., 2016), the factors related to plant-specific characteristics were important too; leaf morphology, for instance, significantly differed among plant species. Leaf morphology of *A. choisyi* (Figure 2) was not as complicated as this of *Gnaphalium* sp. (Figure 3) where the presence of abundant intricate shaped trichomes was distinguishable. Presence of ridges and furrows, veins projections, stomata, cuticular arches, hairs or scales, and sunken position contribute to the leaf roughness (Chaphekar, 2000; Räsänen et al., 2013; Ram et al., 2014). The ridges and grooves enable smaller particles, as well as bacterial cells to get trapped in between. Thus, each plant species retain different amounts and variety of PTE-containing particles on their leaf surfaces, resulting in a kind of selection pressure for the epiphytic bacteria, where only those that are not sensitive to pollution and are likely to obtain nutrients from pollutant dust can remain on the phylloplane. Moreover, other attributes related to nutrients and different elements concentrations, resource availability, defensive compounds, and growth cycles of the host plant affect the functions and composition of the epiphytic community (Yadav et al., 2005; Kembel and Mueller, 2014; Kembel et al., 2014; Thapa et al., 2017), and these should be considered for future studies.

It has to be noted that in the present research, we studied only the cultivable epiphytic community; other complementary approaches can be employed to better understand the dynamic of epiphytic bacteria in a PTE-polluted environment. Currently, our research group is performing automated rDNA intergenic spacer analysis (ARISA) experiments and high throughput Illumina sequencing to investigate the taxonomic and functional diversity of epiphytic microorganisms in polluted environments.

Studies are scarce on phylloremediation involving the major air pollutants (Wei et al., 2017): particulate matters, nitrogen oxides, sulfur dioxide, ground-level ozone, and volatile organic compounds. To the best of our knowledge, this research is a pioneer in the study of epiphytic bacteria from leaves of plants naturally colonizing polluted with PTE (Pb, Cd, Cu, Zn, As, and Sb) in the context of phytoremediation of air pollution. However, we recognize that the composition and functions of the epiphytic community are complex and it is shaped by diverse factors, including environmental conditions, conditions around the leaf, deposition, leaf surface traits, and participation of different groups of microorganisms. The integration of traditional microbiology (cultivable epiphytes) together with cultivation-independent techniques will set the conditions for a better exploration of the interactions between plants and their microbiome inhabiting leaf environment (Müller and Ruppel, 2014) and thus a more profitable implementation of assisted phytoremediation of airborne pollutants.

## Conclusion

The cultivable epiphytic bacterial from the phylloplane of four plant species identified as phytobarriers (*B. veronicifolia*, *F. trinervia*, *Gnaphalium* sp., and *A. choisyi*) and that naturally colonize MR was studied. The leaves of four plant species from MR had higher concentrations of Pb, Zn, As, Cd, and Cu than those from NC. However, no differences were observed in Sb concentration in leaves of *B. veronicifolia*. Plants differed in their role as phytobarriers in the MR according to PTE involved. The highest Zn concentration was observable in leaves of *B. veronicifolia* and *Gnaphalium* sp., and the highest Cu concentration was detected in *F. trinervia*. Despite having a simpler leaf surface in comparison to these in the all three plant species, *A. choisyi* was outstanding species because of its phytobarrier capacity. The highest concentrations of Sb, As, Pb, and Cd were determined in their leaves. Accordingly, the highest amount of CFU per cm^2^ was observed in its leaves collected in MR. Moreover, the number of CFU was positively correlated to the concentration of Cu, Pb, As, Sb, and Cd in leaves. We also analyzed the functional characteristics of morphologically different cultivable bacteria isolated either from PTE-contaminated and NC. Tolerance to PTE (Zn, Pb, Cd, and Cu), tolerance to UV light (up to 12 h of exposition) and drought stress (up to 8 weeks) were functional traits more commonly observed in MR leaf isolates. However, NC isolates were not exempt of having one from the mentioned characteristics. Additionally, more than the half of epiphytic bacteria of *F. trinervia*, and one-third of the isolates of *B. veronicifolia*, *A. choisyi*, and *Gnaphalium* sp., all from in MR, showed phosphate solubilization capacity. These results suggest that the epiphytic cultivable community of plants growing on polluted MR is adapted to the restrictive environmental conditions observed in that kind of sites and this microbial community may support its host plant during the natural colonization of contaminated sites. Moreover, tolerance to PTE, UV, and drought represents an advantage for the plants hosting bacterial epiphytes holding such characteristics. Cultivable epiphytic bacteria from leaves of the studied plant species can be potentially exploited for the phytoremediation of airborne pollutants, especially particulate matter and their associated PTE. Besides the cultivable epiphytic community, molecular approaches should be employed to better understand the dynamic of epiphytic bacteria in a PTE-polluted environment.

## Author Contributions

The experimental performance and elaboration of this manuscript was possible only through the input of different research fields. MG-C and FS-D thoroughly added their profound knowledge about plant–microbe interactions during phytoremediation of PTE. RC-G contributed with his expertise in the behavior and fate of metals in the environment. GR-S provided SEM facilities, technical and operational guidance, and performed images acquirement. AS-L designed and performed the experiments, and the evaluation of results. All authors read and approved the final version of the manuscript to be published.

## Conflict of Interest Statement

The authors declare that the research was conducted in the absence of any commercial or financial relationships that could be construed as a potential conflict of interest.
