# Supplementary material for: Leaf Epiphytic Bacteria of Plants Colonizing Mine Residues: Possible Exploitation for Remediation of Air Pollutants
Source: Front Microbiol. 2018 Dec 7;9:3028. doi: 10.3389/fmicb.2018.03028 (PMC6292962; doi:10.3389/fmicb.2018.03028)
Supplement: Supplementary file 1 [file Table_1.DOCX]

Supplementary Material

Leaf epiphytic bacteria of plants colonizing mine residues: possible exploitation for remediation of air pollutants

Sánchez-López Ariadna S., González-Chávez, Ma. del Carmen. A^2*^, Solís-Domínguez Fernando A., Carrillo-González Rogelio, Rosas-Saito Greta H.

*** Correspondence:** Ma. del Carmen A. González Chávez: [carmeng@colpos.mx](mailto:carmeng@colpos.mx)

# Supplementary Figures and Tables

## Supplementary Table 1. Total concentrations (mg kg^-1^) of potentially toxic elements in the rhizosphere of studied plant species.

| **Plant species** | **Condition** | **Cu** | **Zn** | **Pb** | **Cd** | **As** | **Sb** | **Ag** |
| --- | --- | --- | --- | --- | --- | --- | --- | --- |
| *B. veronicifolia* | MR | 989.8+81.2 | 2691.0+72.3 | 977.3+33.0 | 33.2+2.8 | 5372.9+72.4 | 118.1+31.0 | 10.4+1.3 |
|  | NC | 19.6+3.4 | 36.6+3.0 | 415.2+45.6^†^ | 1.9+0.2 | 1099.8+20.1 | 108.1+19.9 | 1.2+0.4 |
| *A. choisyi* | MR | 1074.9+31.1 | 4827.0+29.4 | 988.6+40.0 | 17.6+2.2 | 15256.8+80.2 | 140.3+9.9 | 6.1+1.1 |
|  | NC | 21.4+5.6 | 99.6+7.7 | 135.8+28.8 | 1.7+0.3 | 1365.1+30.6 | 120.2+10.8 | 1.0+0.0 |
| *F. trinervia* | MR | 67.4+9.5 | 5113.5+92.1 | 1025.4+84.1 | 20.5+2.2 | 18715.5+75.4 | 156.6+23.7 | 6.8+0.6 |
|  | NC | 12.6+1.7 | 61.0+6.9 | 243.7+30.0 | 0.5+0.1 | 1984.9+16.4 | 105.5+12.7 | Nd |
| *Gnaphalium* sp. | MR | 744.8+66.9 | 2844.7+82.2 | 1256.9+73.2 | 11.0+2.0 | 21125.4+77.9 | 162.4+11.3 | 6.0+0.9 |
|  | NC | 9.3+1.2 | 16.6+2.9 | 35.7+5.8 | 0.5+0.1 | 1387.8+36.2 | 26.6+2.1 | Nd |
|  |  |  |  |  |  |  |  |  |
| Range | MR | 67.4-1074.9 | 2691.0-5113.5 | 977.3-1256.9 | 11.0-33.2 | 5372.9-21125.4 | 118.1-162.4 | 6.0-10.4 |
| Range | NC | 9.3-21.4 | 16.6-99.6 | 35.7-415.2 | 0.5-1.9 | 1099.8-1984.9 | 26.6-120.2 | Nd-1.2 |

MR: mine residues; NC: non-contaminated control site; Nd: Non-detected.

## Supplementary Table 2. Tolerance to PTE of epiphytic bacterial isolates of four plant species growing in different contamination conditions.

| **Plant species** | **Isolate** | | **Zn**  **(mg L^-1^)** | | |  | **Cu**  **(mg L^-1^)** | |  | **Cd**  **(mg L^-1^)** | |  | **Pb**  **(mg L^-1^)** | | | |  | **Sb**  **(mg L^-1^)** | | | | |
| --- | --- | --- | --- | --- | --- | --- | --- | --- | --- | --- | --- | --- | --- | --- | --- | --- | --- | --- | --- | --- | --- | --- |
|  |  |  | **40** | **70** | **150** |  | **25** | **50** |  | **45** | **90** |  | **80** | **150** | | |  | **5** | **10** | | **30** | **60** |
| *B. veronicifolia* | MR | BA1 | + | - | - |  | - | - |  | + | - |  | + | - | | |  | + | + | | + | - |
|  |  | BA2 | + | - | - |  | - | - |  | - | - |  | - | - | | |  | + | + | | - | - |
|  |  | BA3 | + | - | - |  | + | - |  | - | - |  | + | - | | |  | + | + | | + | - |
|  |  | BA5 | + | - | - |  | + | - |  | - | - |  | + | - | | |  | - | - | | - | - |
|  |  | BA6 | + | + | + |  | + | - |  | - | - |  | + | + | | |  | + | + | | + | - |
|  |  | BA7 | + | + | + |  | + | - |  | - | - |  | + | + | | |  | + | - | | - | - |
|  |  | BA10 | + | + | - |  | + | - |  | - | - |  | + | - | | |  | - | - | | - | - |
|  |  | BA14 | + | + | - |  | - | - |  | - | - |  | + | - | | |  | + | + | | + | - |
|  |  | BA15 | + | + | + |  | + | - |  | - | - |  | + | - | | |  | + | + | | + | - |
|  |  | BA16 | + | + | + |  | + | - |  | - | - |  | + | - | | |  | + | - | | - | - |
|  |  |  |  |  |  |  |  |  |  |  |  |  |  |  | | |  |  |  | |  |  |
|  | NC | BN1 | + | - | - |  | + | - |  | - | - |  | - | - | | |  | + | + | | - | - |
|  |  | BN2 | + | - | - |  | - | - |  | - | - |  | + | - | | |  | + | + | | + | - |
|  |  | BN3 | - | - | - |  | - | - |  | - | - |  | + | - | | |  | + | - | | - | - |
|  |  | BN4 | - | - | - |  | - | - |  | - | - |  | - | - | | |  | + | - | | - | - |
|  |  | BN5 | + | + | + |  | + | - |  | - | - |  | - | - | | |  | - | - | | - | - |
|  |  | BN7 | + | - | - |  | - | - |  | + | - |  | - | - | | |  | - | - | | - | - |
|  |  | BN10 | + | - | - |  | - | - |  | - | - |  | - | - | | |  | - | - | | - | - |
|  |  |  |  |  |  |  |  |  |  |  |  |  |  |  | | |  |  |  | |  |  |
| *A. choisyi* | MR | DA4 | + | - | - |  | + | + |  | - | - |  | + | + | | |  | + | + | | - | - |
|  |  | DA6 | + | + | + |  | + | + |  | + | - |  | - | - | | |  | + | - | | - | - |
|  |  | DA8 | + | + | + |  | + | - |  | - | - |  | + | - | | |  | + | + | | - | - |
|  |  | DA9 | + | - | - |  | - | - |  | - | - |  | + | - | | |  | + | + | | + | - |
|  |  | DA11 | + | + | - |  | + | - |  | - | - |  | - | - | | |  | + | + | | + | - |
|  |  | DA12 | + | + | - |  | + | - |  | - | - |  | + | - | | |  | + | + | | - | - |
|  |  | DA15 | + | + | + |  | + | + |  | + | - |  | + | - | | |  | + | + | | + | - |
|  |  | DA16 | + | - | - |  | - | - |  | - | - |  | + | + | | |  | + | + | | + | - |
|  |  | DA17 | - | - | - |  | - | - |  | - | - |  | + | - | | |  | + | - | | - | - |
|  |  |  |  |  |  |  |  |  |  |  |  |  |  |  | | |  |  |  | |  |  |
|  | NC | DN1 | + | + | - |  | - | - |  | - | - |  | - | - | | |  | + | + | | + | - |
|  |  | DN4 | - | - | - |  | + | - |  | + | - |  | - | - | | |  | + | + | | + | - |
|  |  | DN5 | - | - | - |  | - | - |  | - | - |  | + | - | | |  | + | + | | - | - |
|  |  | DN7 | + | + | + |  | + | - |  | - | - |  | + | - | | |  | + | + | | + | - |
|  |  | DN8 | - | - | - |  | - | - |  | - | - |  | - | - | | |  | + | + | | - | - |
|  |  | DN11 | + | + | + |  | - | - |  | + | - |  | - | - | | |  | + | + | | + | - |
|  |  | DN15 | - | - | - |  | - | - |  | - | - |  | - | - | | |  | + | + | | - | - |
|  |  | DN16 | + | + | + |  | + | - |  | - | - |  | + | - | | |  | + | + | | - | - |
|  |  |  |  |  |  |  |  |  |  |  |  |  |  |  | | |  |  |  | |  |  |
| *F. trinervia* | MR | FA2 | + | + | - |  | + | - |  | - | - |  | + | - | | |  | + | + | | - | - |
|  |  | FA3 | - | - | - |  | + | - |  | - | - |  | + | - | | |  | + | + | | - | - |
|  |  | FA5 | - | - | - |  | + | - |  | - | - |  | + | - | | |  | - | - | | - | - |
|  |  | FA6 | + | - | - |  | + | - |  | - | - |  | + | - | | |  | + | + | | + | - |
|  |  | FA7 | - | - | - |  | - | - |  | - | - |  | - | - | | |  | - | - | | - | - |
|  |  | FA8 | + | + | - |  | + | - |  | + | - |  | + | + | | |  | + | + | | - | - |
|  |  | FA11 | + | - | - |  | - | - |  | - | - |  | - | - | | |  | + | - | | - | - |
|  |  | FA12 | + | - | - |  | - | - |  | + | - |  | + | - | | |  | - | - | | - | - |
|  |  | FA13 | + | - | - |  | + | - |  | + | - |  | - | - | | |  | + | + | | + | - |
|  |  | FA15 | + | + | - |  | - | - |  | - | - |  | - | - | | |  | + | - | | - | - |
|  |  |  |  |  |  |  |  |  |  |  |  |  | Table continues.. | | | | | | | | | |
|  |  |  |  |  |  |  |  |  |  |  |  |  |  | | | | | | | | | |
|  |  |  |  |  |  |  |  |  |  |  |  |  | Table continuation | | | | | | | | | |
| **Plant species** | **Isolate** | | **Zn**  **(mg L^-1^)** | | |  | **Cu**  **(mg L^-1^)** | |  | **Cd**  **(mg L^-1^)** | |  | **Pb**  **(mg L^-1^)** | | | |  | **Sb**  **(mg L^-1^)** | | | | |
|  |  |  | **40** | **70** | **150** |  | **25** | **50** |  | **45** | **90** |  | **80** | **150** | | |  | **5** | **10** | | **30** | **60** |
| *F. trinervia* | MR | FA17 | + | + | + |  | + | - |  | + | - |  | + | | - |  | | + | | - | - | - |
|  |  | FA19 | + | + | - |  | + | - |  | - | - |  | - | - | | |  | - | - | | - | - |
|  |  |  |  |  |  |  |  |  |  |  |  |  |  |  | | |  |  |  | |  |  |
|  | NC | FN1 | + | + | + |  | - | - |  | - | - |  | + | - | | |  | + | + | | + | - |
|  |  | FN2 | - | - | - |  | - | - |  | - | - |  | + | - | | |  | + | + | | - | - |
|  |  | FN4 | - | - | - |  | - | - |  | - | - |  | + | - | | |  | + | - | | - | - |
|  |  | FN5 | - | - | - |  | - | - |  | - | - |  | - | - | | |  | + | + | | - | - |
|  |  | FN6 | + | + | - |  | - | - |  | + | - |  | - | - | | |  | + | + | | - | - |
|  |  | FN8 | + | + | + |  | + | - |  | + | - |  | + | - | | |  | + | + | | - | - |
|  |  | FN9 | - | - | - |  | + | - |  | - | - |  | - | - | | |  | + | - | | - | - |
|  |  | FN10 | + | - | - |  | + | - |  | - | - |  | + | - | | |  | + | + | | - | - |
|  |  |  |  |  |  |  |  |  |  |  |  |  |  |  | | |  |  |  | |  |  |
| *Gnaphalium* sp. | MR | GA1 | + | + | - |  | + | - |  | - | - |  | + | - | | |  | + | + | | + | - |
|  |  | GA2 | + | + | + |  | + | + |  | - | - |  | + | + | | |  | + | + | | - | - |
|  |  | GA3 | - | - | - |  | - | - |  | - | - |  | - | - | | |  | + | + | | + | - |
|  |  | GA4 | + | - | - |  | - | - |  | + | + |  | + | + | | |  | + | - | | - | - |
|  |  | GA5 | + | - | - |  | - | - |  | + | - |  | - | - | | |  | + | - | | - | - |
|  |  | GA6 | + | + | - |  | - | - |  | + | + |  | - | - | | |  | + | + | | - | - |
|  |  | GA8 | + | - | - |  | - | - |  | + | - |  | + | - | | |  | - | - | | - | - |
|  |  | GA9 | + | - | - |  | - | - |  | + | - |  | + | - | | |  | + | - | | - | - |
|  |  | GA10 | + | - | - |  | - | - |  | + | - |  | + | - | | |  | + | + | | - | - |
|  |  | GA12 | + | - | - |  | - | - |  | - | - |  | - | - | | |  | + | - | | - | - |
|  |  |  |  |  |  |  |  |  |  |  |  |  |  |  | | |  |  |  | |  |  |
|  | NC | GN2 | + | - | - |  | - | - |  | - | - |  | - | - | | |  | - | - | | - | - |
|  |  | GN3 | + | + | + |  | + | - |  | + | - |  | + | + | | |  | + | + | | + | + |
|  |  | GN6 | - | - | - |  | - | - |  | - | - |  | - | - | | |  | + | - | | - | - |
|  |  |  |  |  |  |  |  |  |  |  |  |  |  |  | | |  |  |  | |  |  |

+: growth compared to non-metal supplemented control cultures; -: no growth; MR: Mine Residues; NC: non-contaminated control site.

## Supplementary Table 3. Functional traits of epiphytic bacterial isolates.

| **Plant species** | **Condition** | **Isolate** | **AIA**  **Production** | **N_2_**  **fixation** | **PO^-^_4_**  **solubilization** | **UV tolerance**  **(hours)** | **Drought tolerance**  **(weeks)** |
| --- | --- | --- | --- | --- | --- | --- | --- |
| ***B. veronicifolia*** | MR | BA1 | - | - | + | 0.5 | 2 |
|  |  | BA2 | - | - | - | 0.5 | 2 |
|  |  | BA3 | + | + | - | 12 | 2 |
|  |  | BA5 | - | - | - | 3 | 8 |
|  |  | BA6 | - | - | - | 12 | 6 |
|  |  | BA7 | - | - | + | 0.5 | 6 |
|  |  | BA10 | - | - | - | 1 | 8 |
|  |  | BA14 | - | - | - | 0 | 0 |
|  |  | BA15 | - | - | + | 12 | 8 |
|  |  | BA16 | - | - | - | 0.5 | 0 |
|  |  |  |  |  |  |  |  |
|  | NC | BN1 | + | + | - | 0.5 | 2 |
|  |  | BN2 | - | - | - | 12 | 2 |
|  |  | BN3 | + | + | + | 1 | 8 |
|  |  | BN4 | - | - | - | 0 | 0 |
|  |  | BN5 | - | - | + | 1 | 2 |
|  |  | BN7 | - | - | - | 1 | 6 |
|  |  | BN10 | - | + | - | 0.5 | 4 |
|  |  |  |  |  |  |  |  |
| ***A. choisyi*** | MR | DA4 | - | - | - | 1 | 8 |
|  |  | DA6 | - | - | - | 12 | 6 |
|  |  | DA8 | - | - | - | 0 | 0 |
|  |  | DA9 | - | - | + | 0.5 | 2 |
|  |  | DA11 | - | - | + | 1 | 0 |
|  |  | DA12 | - | - | - | 0 | 0 |
|  |  | DA15 | - | - | - | 0 | 2 |
|  |  | DA16 | + | + | + | 3 | 0 |
|  |  | DA17 | + | - | - | 12 | 2 |
|  |  |  |  |  |  |  |  |
|  | NC | DN1 | + | - | + | 0.5 | 0 |
|  |  | DN4 | - | - | - | 0.5 | 0 |
|  |  | DN5 | - | - | - | 1 | 2 |
|  |  | DN7 | - | - | - | 12 | 8 |
|  |  | DN8 | - | - | + | 0.5 | 4 |
|  |  | DN11 | - | - | - | 0.5 | 2 |
|  |  | DN15 | - | - | - | 0.5 | 8 |
|  |  | DN16 | - | - | - | 1 | 0 |
|  |  |  |  |  |  |  |  |
| ***F. trinervia*** | MR | FA2 | - | - | - | 0.5 | 8 |
|  |  | FA3 | - | + | - | 0 | 2 |
|  |  | FA5 | - | - | + | 0.5 | 2 |
|  |  | FA6 | - | - | + | 0.5 | 6 |
|  |  | FA7 | - | - | - | 0.5 | 6 |
|  |  | FA8 | - | + | - | 0.5 | 2 |
|  |  | FA11 | - | - | + | 1 | 0 |
|  |  | FA12 | - | - | + | 0 | 2 |
|  |  | FA13 | + | + | + | 0 | 0 |
|  |  | FA15 | - | - | + | 1 | 4 |
|  |  | FA17 | - | - | - | 0.5 | 0 |
|  |  | FA19 | + | - | - | 0.5 | 6 |
|  |  |  |  |  |  |  | Table continues |
|  |  |  |  |  |  |  |  |
|  |  |  |  |  |  |  |  |
|  |  |  |  |  |  | Table continuation | |
| **Plant species** | **Condition** | **Isolate** | **AIA**  **Production** | **N_2_**  **fixation** | **PO^-^_4_**  **solubilization** | **UV tolerance**  **(hours)** | **Drought tolerance**  **(weeks)** |
| ***F. trinervia*** | NC | FN1 | - | - | + | 1 | 0 |
|  |  | FN2 | - | - | - | 0 | 0 |
|  |  | FN4 | - | - | - | 0.5 | 0 |
|  |  | FN5 | - | + | - | 0.5 | 2 |
|  |  | FN6 | + | - | - | 1 | 6 |
|  |  | FN8 | - | - | + | 12 | 0 |
|  |  | FN9 | - | - | - | 12 | 8 |
|  |  | FN10 | - | - | - | 1 | 8 |
|  |  |  |  |  |  |  |  |
| ***Gnaphalium* sp.** | MR | GA1 | - | - | + | 12 | 4 |
|  |  | GA2 | - | - | - | 12 | 0 |
|  |  | GA3 | - | - | - | 1 | 0 |
|  |  | GA4 | - | - | + | 0.5 | 0 |
|  |  | GA5 | - | - | - | 1 | 8 |
|  |  | GA6 | - | - | + | 0.5 | 6 |
|  |  | GA8 | - | - | - | 0.5 | 2 |
|  |  | GA9 | - | - | - | 6 | 0 |
|  |  | GA10 | - | + | - | 1 | 8 |
|  |  | GA12 | + | - | - | 0 | 6 |
|  |  |  |  |  |  |  |  |
|  | NC | GN2 | - | - | - | 0.5 | 0 |
|  |  | GN3 | + | - | + | 3 | 2 |
|  |  | GN6 | + | - | - | 0 | 2 |

Indol Acetic Acid (IAA); positive (+) or negative (-) results for test of certain functional trait; MR: Mine Residues; NC: non-contaminated control site.

## Supplementary Table 4. Significant correlations among evaluated variables.

|  | **Sb-W** | **Zn-NW** | **Pb-NW** | **Cd-NW** | **As-NW** | **Sb-NW** | **Zn-S** | **Pb-S** | **Cd-S** | **As-S** | **Chl T** | **CFU** | **H** |
| --- | --- | --- | --- | --- | --- | --- | --- | --- | --- | --- | --- | --- | --- |
| **Zn-W** | 0.74** | 0.76** | 0.70* | 0.70* | 0.76** | 0.70* |  | 0.70* |  | 0.71* |  |  |  |
| **Pb-W** | 0.71* | 0.83** | 0.70* | 0.70* | 0.75** |  | 0.71* | 0.71** |  | 0.70* |  |  | -0.70** |
| **As-W** | 0.71** | 0.72** |  |  |  |  |  |  |  |  | 0.70* |  | -0.71* |
| **Sb-W** |  |  | 0.75** | 0.78** | 0.72* | 0.70* |  |  | 0.73* | 0.72* |  | 0.71* |  |
| **Cu-NW** |  | 0.70* | 0.95** | 0.95** | 0.89** | 0.93** |  | 0.85** | 0.73* | 0.83** |  | 0.77* |  |
| **Zn-NW** |  |  | 0.70* | 0.70* |  |  | 0.85** | 0.71** |  | 0.82** |  |  |  |
| **Pb-NW** |  |  |  | 0.89** | 0.92** | 0.91** |  | 0.96** | 0.70* | 0.88** |  | 0.70* |  |
| **Cd-NW** |  |  |  |  | 0.85** | 0.92** |  | 0.82** | 0.72* | 0.82** |  | 0.85** |  |
| **As-NW** |  |  |  |  |  |  |  | 0.91** |  |  |  | 0.70* |  |
| **Sb-NW** |  |  |  |  |  |  |  |  |  |  |  | 0.75* |  |
| **Pb-S** |  |  |  |  |  |  |  |  |  | 0.86** |  | 0.70* |  |
| **Cd-S** |  |  |  |  |  |  |  |  |  |  |  | 0.83** |  |
| **Chl_a** |  |  |  |  |  |  |  |  |  |  | 0.87** |  |  |
| **Chl_b** |  |  |  |  |  |  |  |  |  |  | 0.72* |  | -0.70* |
| **Chl_T** |  |  |  |  |  |  |  |  |  |  |  |  | -0.71* |

W: concentration of certain element in washed leaf samples; NW: concentration of certain element in non-washed leaf samples; S: concentration of certain element on leaf surfaces; Chl T: concentration of total chlorophyll; Chl a: concentration of chlorophyll a; Chl_b: concentration of chlorophyll b; CFU: number of epiphytic bacterial colonies forming units; H: Shannon diversity index; * significant correlation (p<0.05); ** highly significant correlation (p<0.001) according to Spearman´s rank test.
